# Influence of Stimulant Medication and Response Speed on Lateralization of Movement-Related Potentials in Attention-Deficit/Hyperactivity Disorder

**DOI:** 10.1371/journal.pone.0039012

**Published:** 2012-06-14

**Authors:** Stephan Bender, Franz Resch, Christoph Klein, Tobias Renner, Andreas J. Fallgatter, Matthias Weisbrod, Marcel Romanos

**Affiliations:** 1 Section for Clinical Neurophysiology and Multimodal NeuroImaging, Child and Adolscent Psychiatric Hospital of the University of Technology, Dresden, Germany; 2 Department of Child and Adolescent Psychiatry, Ruprecht Karls University, Heidelberg, Germany; 3 School of Psychology, University of Bangor, Bangor, Wales, United Kingdom; 4 Department of Child and Adolescent Psychiatry, Psychosomatics and Psychotherapy, University of Würzburg, Würzburg, Germany; 5 Department of Psychiatry, University of Tübingen, Tübingen, Germany; 6 Section for Experimental Psychopathology, Psychiatric Hospital of the University of Heidelberg, Heidelberg, Germany; 7 Psychiatric Hospital, SRH, Karlsbad-Langensteinbach, Germany; 8 Department of Child and Adolescent Psychiatry, Psychosomatics and Psychotherapy, University of Munich, Munich, Germany; University of Wuerzburg, Germany

## Abstract

**Background:**

Hyperactivity is one of the core symptoms in attention deficit hyperactivity disorder (ADHD). However, it remains unclear in which way the motor system itself and its development are affected by the disorder. Movement-related potentials (MRP) can separate different stages of movement execution, from the programming of a movement to motor post-processing and memory traces. Pre-movement MRP are absent or positive during early childhood and display a developmental increase of negativity.

**Methods:**

We examined the influences of response-speed, an indicator of the level of attention, and stimulant medication on lateralized MRP in 16 children with combined type ADHD compared to 20 matched healthy controls.

**Results:**

We detected a significantly diminished lateralisation of MRP over the pre-motor and primary motor cortex during movement execution (initial motor potential peak, iMP) in patients with ADHD. Fast reactions (indicating increased visuo-motor attention) led to increased lateralized negativity during movement execution only in healthy controls, while in children with ADHD faster reaction times were associated with more positive amplitudes. Even though stimulant medication had some effect on attenuating group differences in lateralized MRP, this effect was insufficient to normalize lateralized iMP amplitudes.

**Conclusions:**

A reduced focal (lateralized) motor cortex activation during the command to muscle contraction points towards an immature motor system and a maturation delay of the (pre-) motor cortex in children with ADHD. A delayed maturation of the neuronal circuitry, which involves primary motor cortex, may contribute to ADHD pathophysiology.

## Introduction

As hyperactivity is one of the core symptoms of attention deficit hyperactivity disorder (ADHD), it is crucial to understand the role of the motor system in the disease. Structural magnetic resonance imaging (MRI) studies and recent functional imaging data indicate that a disturbance of motor function in the primary motor cortex might contribute to ADHD pathophysiology [1], [2]. In line with these findings, transcranial magnetic stimulation studies have shown decreased inhibition in the motor system of children [3], [4] and adults [5] with ADHD. Movement-related potentials (MRP) offer the possibility to separate various functionally different movement stages and display a sequence of components that can be attributed to movement initiation and early preparation (Bereitschaftspotential), advanced movement programming and execution (initial motor potential peak; iMP) and post-processing (motor postimperative negative variation; mPINV) with distinct topographical features and lateralization [6], [7]. The assessment of lateralized potentials allows evaluating movement “selection” and focal cortical activation instead of general “force” aspects [8]. While iMP is related to the sending of the cortico-spinal volley which leads to muscle contraction, mPINV is thought to reflect a short term memory trace in the motor system [6], [9], [10]. During brain maturation in childhood and adolescence, the Bereitschaftspotential and the iMP change polarity from a positive to a negative potential around 10–12 years [11], [12], [13], [14]. In contrast, mPINV shows decreasing negative amplitudes, i.e. younger children have the largest negative peaks [15]. Movement-related potential studies in ADHD so far have mainly focused on contingent negative variation and response preparation/attentional resource allocation processes [16], [17], [18], [19], [20], [21], [22], [23] and much less on potentials related to movement execution [24], [25] or sensorimotor movement evaluation.

Indirectly, the top-down control of the motor system can be assessed when MRP are examined under specific conditions. An increased number of very slow responses in children with ADHD may reflect more frequent lapses of attention. Thus responses with long reaction times have been used to examine functional states in which the subjects are less concentrated than in trials with short reaction times [26], [27], [28].

Finally, the motor system is crucially influenced by dopamine. MRP could reflect an excellent neurophysiological marker to monitor the effects of stimulant medication in ADHD [29], [30]. During movement execution, lateralized MRP amplitudes (iMP’) seem rather independent of dopaminergic medication [31], [32]. In contrast, mPINV amplitude has been found to be affected by first generation antipsychotics (dopamine antagonists) [33].

In order to characterize how ADHD, response speed (i.e. more or less concentrated states) and stimulant medication would differentially affect the neuronal activation related to triggering a movement (iMP) or its post-processing in short term motor memory (mPINV), we analysed lateralized MRP in a previously characterized sample of children with ADHD and matched controls [34], [35]. Based on the above mentioned literature, we hypothesized that children with ADHD would show less lateralized iMP potentials especially in trials with slow reaction times (lapses of attention). Previous studies of lateralised ERP in ADHD reported reduced contingent negative variation [21] and diminished lateralised MRP preceding the movement [24]. In contrast, increased PINV amplitudes in children with ADHD and tic disorder have been described under conditions of loss or lack of control [16]. Together with a correlation of increased omission errors with larger motor PINV’ amplitudes [36], these findings suggest that reduced attention may be associated with reduced iMP’ (less motor preparation) but increased mPINV’ (compensatory post-processing). Additionally, we hypothesized to find stimulant effects, which would specifically affect mPINV’ but not iMP’ amplitude (because dopaminergic medication in previous studies affected only lateralized post-movement potentials [31], [32]), pointing to medication-related compensation processes.

## Methods

### 2.1 Subjects

Seventeen boys with attention deficit hyperactivity disorder according to the ICD-10 criteria for F90.0 (corresponding to the DSM IV combined type of ADHD) by an interdisciplinary team (mean age 9.5±1.5 years; range 7.2 to 11.7 years; IQ 106±14.1; range 83–121) and 20 age- and IQ-matched healthy control boys (mean age 9.9±1.1 years; range 8.2 to 11.8 years; IQ 111±13.2; range 97–132) were recruited (group means were matched). For the response-locked EEG analysis in the current paper, we had to exclude one ADHD patient due to an insufficient number of successful response trials (N<10). The remaining sample consisted of N = 16 patients (mean age 9.6±1.5 years, IQ 104±14.3). IQ was assessed by the German version of the Wechsler Intelligence Scale for Children (HAWIK III).

Two reports about stimulus-locked data analysis in this sample have been published before [34], [35], however, these reports did not include any movement-related potential analyses. All subjects were right-handed according to the Edinburgh Handedness Inventory [37]. Patients were recruited from the University Hospital Würzburg, Germany. No psychiatric or neurological comorbid disorders were allowed. None of the subjects was taking psychoactive medication except for stimulant medication in the ADHD group. ADHD subjects were tested off-medication and after the intake of 10 mg immediate-release methylphenidate (MPH), i.e. a mean MPH dose of 0.34 mg/kg (range 0.27–0.47 mg/kg). Recordings on and off medication were separated by one week. For 10 ADHD subjects, the off-medication session was conducted first, for 7 children with ADHD the order was reverse. Subjects who were recorded off medication first, were naïve to stimulant medication. Subjects who were recorded on medication first withdrew the medication for one week before the second session [4], [34], [35]. All recordings took place at 9 am, which was one hour after medication intake during the on-medication session of the patient group. For ethical reasons, no MPH was given to healthy control children.

#### 2.1.1 Ethics statement

The study was approved by the ethics committee of the Medical Faculty of the University of Würzburg, Germany. All subjects and their parents provided written informed consent according to the Declaration of Helsinki.

### 2.2 Task

Subjects performed a modified version of the continuous performance test (CPT-OX) [38]. 400 letters were presented for 150 ms at fixed 1,650 ms inter-stimulus intervals. Subjects were required to respond whenever a target letter (“X”) followed a cue letter (“O”). The cue “O” was presented 80 times (20%). The Go-stimulus “X” followed the cue “O” in 50% of the cases and was thus presented 40 times. Another 40 times, the cue “O” was followed by another letter (A, B, C, D, E, F, G, H, J or L; No-Go condition). These ten other letters also served as distractors. Unilateral button press responses by the dominant right hand were required as fast and accurately as possible.

### 2.3 Data Acquisition

EEG was recorded from 21 gold cup electrodes which were placed according to the international 10–20 system using a sampling rate of 256 Hz and a band pass filter of 0.3 to 70 Hz. The current analysis focused on movement-related potential components which are evoked in response to cued reactions (details are given below). These components have a shorter duration [6], [39] than the readiness potential or contingent negative variation (which were not assessed in the current study), so that the attenuation of potentials lasting longer than 2 seconds by the high-pass filter only eliminated possible confounding factors. Linked earlobes served as recording reference. Vertical and horizontal electrooculogram were also registered from electrodes about 1 cm above and below the left eye as well as next to the outer epicanthi. Electrode impedances were maintained below 5 kOhm.

### 2.4 Data Preprocessing

**Figure 1:**
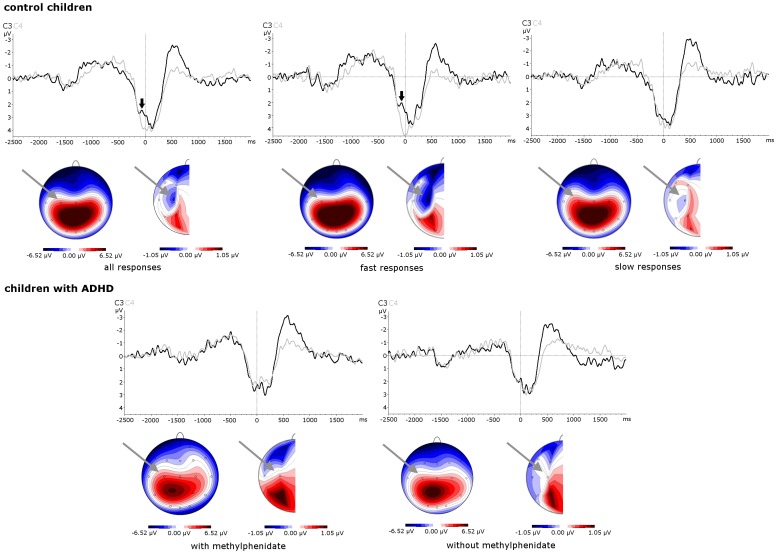
Time-course and topography of response-locked movement-related potentials (initial movement related potential peak - iMP) including the topography of iMP lateralization ([C3−C4]/2). For control children, averages of all responses are illustrated together with a separate presentation of averages of fast (below median reaction time) and slow responses (above median reaction time). For children with ADHD, responses on and off methylphenidate are presented. For effects of response speed in ADHD see Figure 4. Note how the rather symmetrically distributed stimulus-related P300 shadowed MRP in the topography before the subtraction of symmetrically distributed potential components by the calculation of lateralization. iMP time-course (thick black arrows) and lateralized topography around C3 (grey arrows) are in good agreement with previous literature. mPINV (lateralized negativity at C3 in the time interval 500–800 ms) was not shadowed by P300 any more. Its topography is presented in Figure 2.

Only trials with correct responses within 1 second were included in the analysis. Data were segmented on response triggers from −2500 to 2000 ms. The first 200 ms of this epoch served as baseline. Taking into account that median reaction times were about 400–500 ms and that there was an interval of 1800 ms between the onset of the cue ‘O’ and the onset of the the target ‘X’, this baseline fell before the cue for nearly all responses in all subjects when fast reactions below the median were analyzed. We refrained from an even earlier baseline in order to avoid a contamination by preceding responses, though cues were preceded by distractors. We made sure that this baseline was not contaminated by lateralized responses to the cue or late MRP to the preceding trial. In Figure 1 it becomes evident, that the potential time courses are quite parallel and the reported differences refer to specific time intervals relative to the given response.

Data were corrected for ocular artifacts using the algorithm according to Gratton and Coles (BrainVision Analyzer, BrainProducts, Munich, Germany). Artifacts were automatically rejected when the signal amplitude exceeded 150 µV due to the higher background EEG in children compared to adults. This procedure was controlled by visual inspection. The average reference was calculated offline.

### 2.5 Data Analysis

Response locked lateralized MRP were assessed at C3 versus C4 [40], [41], the site of the topographic maximum of movement-related potential lateralization (Figure 1). Though no complete lateralized readiness potential could be calculated because no left hand button presses were available, previous studies have shown that lateralization of MRPs is much stronger for button presses with the dominant hand [40], [41]. We checked carefully in a comparison with stimulus-locked data [34], [35], that the time-course of our reported results differed from the time-course of any possible lateralization of the P300/late positive complex. P300 topography showed a left-lateralized positive maximum with the applied verbal (letter) task and led to a positive peak in P3–P4 (see Figure 1). It could only reduce and not artificially produce lateralized negativity at C3 during the iMP which showed a different time course than P300 (Figure 1). The iMP’ (apostrophes indicate that *lateralization* of iMP or mPINV was assessed) was calculated as the lateralized amplitude in the time window −70 to −20 ms before the response trigger by the formula (C3–C4)/2; the mPINV’ was calculated in the same way as the lateralized amplitude in the time window 500–800 ms after the response trigger, in accordance with previous reports [6].

### 2.6 Statistics

#### 2.6.1 Behavioral data

Group differences in reaction times as well as effects of medication and learning were examined by Student’s t-tests.

#### 2.6.2 Electrophysiological data

Group effects: iMP’ and mPINV’ amplitudes were examined in an ANOVA with the between subject factor GROUP (unmedicated ADHD versus healthy controls) and the repeated measurement factor COMPONENT (iMP’ versus mPINV’). The factor component was introduced to test for differential group effects (interaction GROUP x COMPONENT) on pre- and post-movement potentials as these have been shown to differ in their maturational trajectories [15], to be influenced in different ways by dopaminergic medication [31], [32] and other related evoked potentials (lateralized readiness potential, PINV) have been found to be altered in opposite directions in ADHD [16], [24]. Any significant interactions were confirmed by subsequent univariate analyses. In an additional analysis, we controlled for effects of REACTION TIME using median reaction times as a covariate and checked whether any significant effects would persist when AGE was introduced as another covariate. Significant interactions were further explored by Newman Keuls post-hoc tests.

Effects of stimulant medication (10 mg MPH): iMP’ and mPINV’ amplitudes were examined in an ANCOVA with the repeated measurement factors COMPONENT (iMP’ versus mPINV’) and MEDICATION (on/off methylphenidate). Again, median reaction times (off medication) served as a covariate in an additional analysis. Reaction time off medication was used as covariate instead of the mean between reaction times on and off medication because theoretically, medication effects on reaction time could have masked relevant findings. However, results did not change when the mean value of median reaction times on/off medication was used as a covariate instead of median reaction times off medication (not shown).

Finally, in order to assess the effects of response speed, a median split was performed into trials with slow and fast reaction times. An ANOVA with the between subject factor GROUP (ADHD versus healthy controls) as well as the repeated measurement factors COMPONENT (iMP’ versus mPINV’) and REACTION TIME (below versus above median reaction time) was calculated.

Pearson correlation coefficients between median reaction times and iMP’ amplitudes were calculated. This was done separately for the two diagnostic groups because we obtained group differences for iMP’ amplitudes (see group effects above).

## Results

### 3.1 Behavioural Data

Without medication, ADHD subjects had longer median reaction times compared to healthy control children (498±135 ms vs 421±77 ms; t = 2.2; p = 0.04). The difference was more pronounced for slow responses above (671±204 ms vs. 505±91 ms; t = 3.3; p = 0.002) than fast responses below median reaction time (405±79 ms vs. 359±69 ms; t = 1.9; p = 0.07). Median reaction time was reduced in ADHD subjects under methylphenidate compared to the run without medication (430±69 ms; t = 2.2; p = 0.04).

### 3.2 Electrophysiological Data

#### 3.2.1 Group effects

**Figure 2:**
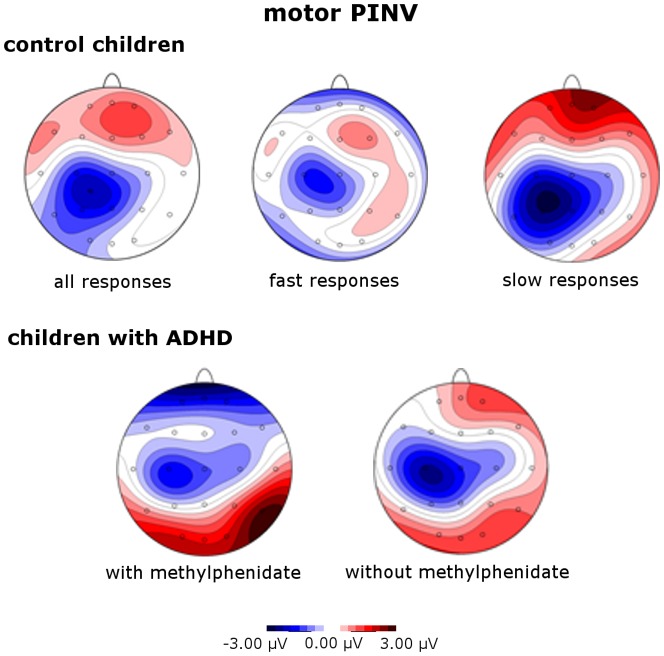
Motor PINV topography for healthy control children. (top; from left to right: all responses, fast responses below median reaction time, slow responses above median reaction time) **and children with ADHD** (bottom; from left to right: responses off and on methylphenidate) for the motor PINV time interval (500–800 ms after the response trigger, motor post-processing).

The time-course of potentials at central leads C3 versus C4 (Figure 1) showed a difference between C3 and C4 right before the response movement during the iMP time interval. Before the subtraction of symmetrically distributed potentials (Figure 1, iMP topography before and after the calculation of lateralized potentials), the P300/late positive complex shadowed movement-related potentials, a usual effect for response movements. Figure 2 illustrates mPINV topography. Mean iMP’ and mPINV’ amplitudes are presented in Table 1 in order to further illustrate lateralization (supplementary Table S1 provides values at C3/C4 separately).

**Table 1:** Lateralized motor potential (iMP’) and motor postimperative negative variation (mPINV’) amplitudes for the ADHD and the healthy control group ([C3**−**C4]/2).

	ADHD	control children
	mean ± standarderror (SE)	mean ± SE
iMP’ (without MPH)	0.29±0.25 µV	−0.64±0.23 µV
iMP’ (with MPH)	0.03±0.33 µV	
mPINV’ (without MPH)	−0.78±0.21 µV	−0.67±0.18 µV
mPINV’ (with MPH)	−0.44±0.28 µV	

There was a main effect of COMPONENT as well as an interaction between diagnostic GROUP and COMPONENT in the ANOVA with these two factors (see Table 2) when iMP’ and mPINV’ amplitudes of unmedicated children with ADHD were compared to typically developing children. Diagnostic GROUP did not reach the significance level for a main effect (Table 2).

**Table 2:** ANOVA results (comparison children with ADHD versus healthy control children).

a) between-subject factor GROUP, repeated measurement factor COMPONENT			
GROUP	F(1;34) = 3.4	p = 0.07	
COMPONENT	F(1;34) = 6.4	p = 0.02	
GROUP x COMPONENT	F(1;34) = 5.6	p = 0.02	
**b) univariate ANOVAs factor GROUP for both components separately**			
iMP’:	GROUP	F(1;34) = 7.6	p = 0.009
mPINV:	GROUP	F(1;34) = 0.1	p = 0.72

Controlling for REACTION TIME as a covariate, the factors diagnostic GROUP and COMPONENT still interacted (F(1;33) = 4.9; p = 0.03). Newman Keuls post hoc tests showed that iMP’ amplitude was reduced in children with ADHD (p = 0.005; cf. Table 1). However, mPINV’ amplitudes did not differ (p = 0.74). As a consequence, ADHD children showed higher mPINV’ than iMP’ amplitudes (p = 0.009; Newman Keuls post hoc test) while healthy controls did not (p = 0.91). Adding AGE as another covariate in addition to the between subject factor GROUP and the repeated measurement factor COMPONENT did not change these results (interaction GROUP x COMPONENT F(1;32) = 4.5; p = 0.04).

There were no significant differences in iMP’ amplitude between medication naïve children who were tested off medication first and children who had been medicated before and were tested on medication afterwards (t = 0.4; p = 0.68; unpaired t-test).

Stimulus locked waveforms are given for comparison in supplementary Figure S1.

#### 3.2.2 Medication effects (children with ADHD only)

**Figure 3:**
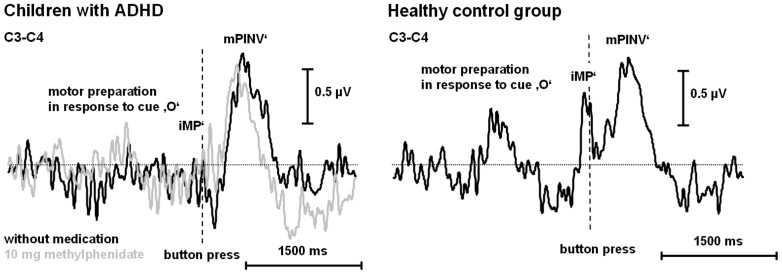
Group effects and effects of stimulant medication. Grand average of lateralized motor potentials ([C3**−**C4]/2) for children with ADHD and healthy control children. In ADHD children, the potential waveform before and after intake of 10 mg methylphenidate are shown – the order in which the sessions were recorded was counterbalanced.

The ANOVA with the factors MEDICATION and COMPONENT did not yield any significant main effect or interaction (Table 3). However, controlling for REACTION TIME as a covariate, subjects with ADHD showed an interaction between the factors COMPONENT and MEDICATION (F(1;14) = 5.8; p = 0.03). The difference between iMP’ and mPINV’ amplitudes (without medication p = 0.004) was reduced under MPH (on medication p>0.05), because mPINV tended to show reduced amplitudes (Table 1) due to an earlier rise and a shorter duration (Figure 3). Nevertheless, since there was no negative potential during the iMP’ in the medicated condition in children with ADHD, MPH did not lead to normalization of iMP’ amplitudes (Table 1 and Figure 3).

**Table 3:** ANOVA results (effects of methylphenidate).

a) Repeated measurement factors COMPONENT and MEDICATION:		
COMPONENT	F(1;15) = 3.1	p = 0.096
MEDICATION	F(1;15) = 0.0	p = 0.90
COMPONENT × MEDICATION	F(1;15) = 2.6	p = 0.14
**b) Univariate ANOVAs factor COMPONENT for patients on/off medication:**		
off medication:	F(1;15) = 9.1	p = 0.009
on medication:	F(1;15) = 0.7	p = 0.42

#### 3.2.3 Effects of response speed: Median split according to reaction times

**Figure 4:**
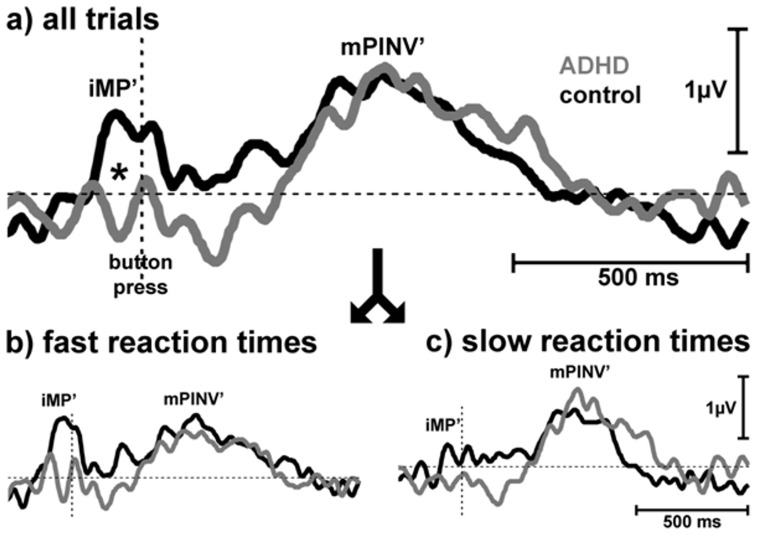
Effects of response speed. Grand average of lateralized motor potentials ([C3**−**C4)/2] of a) all responses time-locked to motor response; *: p<0.05; b) fast responses below median reaction time; c) slow responses above median reaction time. Lateralisation of the initial motor potential peak (iMP) was diminished in ADHD children especially during short, but not during long reaction time.

Response speed affected the group differences in MRP amplitudes (interaction GROUP x RESPONSE SPEED F(1;34) = 4.4; p = 0.04). Group differences were present only for fast reaction times below the median (p = 0.045) but not for slow reaction times above the median (p = 0.67; Newman Keuls post hoc tests; Figure 4). Trend level for an interaction RESPONSE SPEED x COMPONENT (F(1;34) = 2.7; p = 0.11) was not reached.

**Figure 5:**
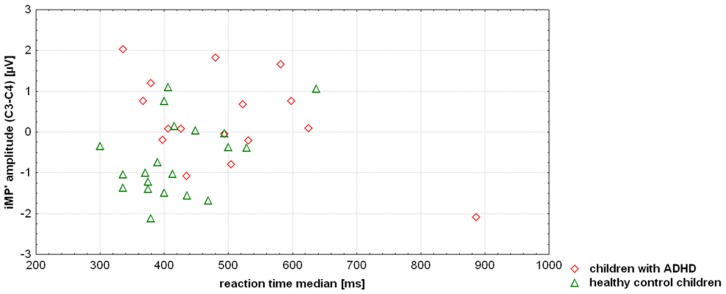
Scatterplot showing the correlation between response speed and lateralized initial motor potential peak amplitude (iMP’) for children with ADHD and healthy control children ([C3−C4]/2).

There was a trend towards a positive correlation between median reaction times and iMP’ amplitude in healthy controls (r = 0.43; t = 2.0; p = 0.06) but a negative correlation in the ADHD group (r = −0.50; t = 2.2; p = 0.048; Figure 5). The two correlations differed significantly (F(1;32) = 8.2; p = 0.007). Though there was one subject with a high reaction time median in the ADHD group, the results of the median split analysis given above supported the interpretation that the regression differences were not artificially caused by this outlier.

## Discussion

To our knowledge, this is the first study, which examines the influence of response speed and medication on movement-related potentials in ADHD, separating pre- and post-movement processing. Contralateral focal activation of the premotor and primary motor cortex during motor response programming (iMP’) exhibited a significantly reduced lateralization in children with ADHD compared to healthy controls, especially when reaction times were short. For the motor memory trace (mPINV’) this effect was not found, mPINV’ amplitude was even non-significantly higher (i.e. more negative) in trials with slow responses. Unlike control children, unmedicated children with ADHD showed significantly larger mPINV’ than iMP’ amplitudes. While in healthy controls shorter reaction times were associated with more negative iMP’ amplitudes, this pattern was reversed or at least absent in ADHD subjects. This finding appears plausible as several structural [42], [43] and functional findings [44], [45] point towards maturation delays in ADHD. In younger children, iMP amplitudes still show a positive polarity, while polarity changes in adolescents [11], [12], [13], [14], [46]. Thus the inverse association between reaction time and iMP’ amplitude in children with ADHD compared to healthy control children may point towards the interpretation that reduced iMP’ amplitudes in children with ADHD in our sample may result from a delayed motor cortex maturation. The fact that children with ADHD showed a more negative mPINV’ than iMP’ amplitude fits to this idea, as this pattern is usually found in younger children [15]. A delayed maturation would also explain why the results contradicted our initial hypothesis: If in ADHD fast responses are associated with more positive potentials and in healthy control children fast responses are associated with more negative potentials, differences will be largest in fast response trials even though lapses of attention may occur more often in subjects with ADHD. A delayed maturation could also explain why MPH did not normalize movement-related potentials completely.

Short reaction times may be taken as indication of better concentration on the task. Movement kinetics themselves (speed of the movement, muscle force) have been found to be largely independent of lateralized MRP amplitudes [8], [32], [47], [48], [49]. Thus, differences in the motor cortex recruitment by higher cortical areas (indicated by differences in response speed [50]) may have crucially influenced iMP’ amplitude but could not account for all the diagnosis-related deficits because ADHD children did not present negative iMP’ amplitudes even for fast responses.

Previous studies of lateralised ERP in ADHD support our findings and have shown a reduced contingent negative variation [21] and diminished lateralised ERP within a time range compatible to the iMP [24]. However, this was not a unanimous finding, as the lateralised readiness potential was not reduced in all studies [25]. fMRI research has reported reduced activity of the contralateral motor cortex in ADHD in a finger-tapping task [1]. Increased PINV amplitudes in children with ADHD and tic disorder have been described under conditions of loss or lack of control [16]. Our study further refines these findings and contributes to separating motor [6], [39] and cognitive PINV components [51], [52], [53].

Methylphenidate tended to normalize prolonged response latencies in ADHD children like in previous studies [54]. MPH also tended to normalize lateralized movement-related potentials in agreement with previous literature [55] as it led to a significant reduction of the difference between mPINV’ and iMP’ amplitude in children with ADHD. The interaction between COMPONENT and MEDICATION only reached significance level when we controlled for reaction time. The inclusion of reaction time as a covariate did not make a big difference (the p-value of the interaction was p = 0.14 without and p = 0.03 with reaction time as a covariate). However, even so, the fact that the statistical significance of the COMPONENT x MEDICAT’ION effect depended on the inclusion of reaction time as a covariate could indicate that the interaction effect was caused by either the reaction time or by the medication effect. This confounding effect could not be resolved statistically. A reduction of iMP’ and mPINV’ differences under MPH would be in line with findings in a different sample showing that dopamine affects pre- and post-movement potentials in a different manner, with distraction leading to lower pre- and higher post-movement processing [36]. First generation antipsychotics (dopamine antagonists) and Parkinsons’s disease also affect pre- and post-movement potentials in a different way [39]. However, even under MPH children with ADHD did not present negative iMP’ amplitudes. Additionally, a reduction of mPINV’ latency under MPH becomes evident in Figure 1 and may point towards medication related changes in response movement kinetics rather than a true mPINV’ amplitude reduction.

### 4.1 Maturational Changes in the Motor Cortex in ADHD

Taken together, these findings indicate a qualitative difference in focal motor cortex activation in ADHD, which cannot be compensated for by medication or top-down control when only trials with short reaction times are taken into account. Previous studies have repeatedly shown a polarity reveral during childhood [11], [12], [14], [45] and explanations for the change in pre-movement MRP polarity during childhood have referred to a substitution of axodendritic by axosomatic synapses in the primary motor cortex [14]. The polarity of a surface EEG potential is determined by the depth of the postsynaptic potentials in the cortex: Excitatory activity in superficial (apical dendrites) and inhibitory activity in deeper layers (cell somata) produce surface negativity. Qualitative changes in primary motor cortex seem more likely than increased inhibition of unwanted movements in younger children [46]. Younger children show an event-related desynchronization in the alpha band during response preparation like adolescents do [11]. Moreover, transcranial magnetic stimulation studies point towards a disinhibition instead of increased inhibition during response preparation also in 6–10 year-old children [56]. These facts show that a polarity reversal of movement-related potentials due to maturational changes in the cortex appear more likely than other explanations. There was a largely symmetric time-course at C3 and C4 except for the iMP’ and mPINV time intervals in fast responses of healthy control children (Figure 1). The potential at C3 during the iMP was more negative compared to C4 in (fast responding) healthy children while children with ADHD show a rather symmetric positivity at C3 and C4. Taken into account that this deviation of C3 from C4 specifically affected the iMP interval right before movement onset (Figure 1), the most likely explanation is a true negative potential in C3. However, due to the overlap with the P300 complex, we cannot completely exclude contributions from positive potentials at C4.

The identification of lateralized movement-related potentials with their characteristic time course [6], their specific modulation by stimulant medication and the absence of a baseline contamination provide strong support for the assumption that the previously reported diminished P300 [35], [57] did not contribute to our results.

We would like to emphasize, that ADHD is not a simple maturation delay, as findings about differences which persist into adulthood demonstrate. Some aspects of maturation seem to be delayed in ADHD and may contribute to (though not fully explain) ADHD pathology [58].

A limitation of our study is the small sample size and that data from a continuous performance test were re-analyzed. The fact that subjects had to be prepared to inhibit their responses in some trials may have influenced our results. Future studies should include a standardized characterization of clinical motor problems in the examined sample and employ a wider range of motor paradigms ranging from freely selected spontaneous movements to pre-programmed movement sequences.

### 4.2 Conclusions

Response speed crucially modulates lateralized MRP amplitudes. Surprisingly, the most pronounced differences between ADHD and healthy control children were found in trials with fast reaction times, i.e. good concentration. The inverse association of response speed and iMP’ amplitude in the control and the ADHD group pointed towards a maturation delay in the motor system of ADHD children in our sample. Stimulant medication tended to normalize response speed, but did not normalize iMP’ amplitudes, giving further support to the hypothesis that the substitution of axodendritic by axosomatic synapses may be delayed in the motor cortex in ADHD children. This hypothesis warrants further investigation.

## Supporting Information

Figure S1 **Stimulus-locked lateralized potentials ([C3−C4)/2).** There was a negative peak in healthy control children with a latency of about 300 ms. Most likely, it corresponds to the response-locked iMP’ in fast responses (latency about 360 ms). In ADHD children, even taking into account their slightly longer reaction times, no corresponding peak could be found. Stimulus locked lateralized potentials should be interpreted with caution due to possible confounding effects of P300/late positive complex.
(TIF)

Table S1 **Data before the calculation of lateralization (mean ± standard error).**
(DOC)
