# Supplementary figures and images for: Influence of Stimulant Medication and Response Speed on Lateralization of Movement-Related Potentials in Attention-Deficit/Hyperactivity Disorder
Source: PLoS One. 2012 Jun 14;7(6):e39012. doi: 10.1371/journal.pone.0039012 (PMC3375242; doi:10.1371/journal.pone.0039012)

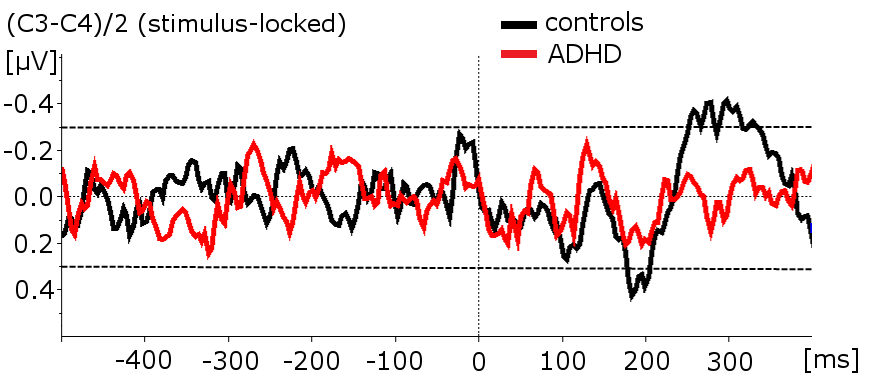

Supplement: Figure S1 — Stimulus-locked lateralized potentials ([C3−C4)/2). There was a negative peak in healthy control children with a latency of about 300 ms. Most likely, it corresponds to the response-locked iMP’ in fast responses (latency about 360 ms). In ADHD children, even taking into account their slightly longer reaction times, no corresponding peak could be found. Stimulus locked lateralized potentials should be interpreted with caution due to possible confounding effects of P300/late positive complex. (TIF) [file pone.0039012.s001.tif]
