# Supplementary material for: Influence of Stimulant Medication and Response Speed on Lateralization of Movement-Related Potentials in Attention-Deficit/Hyperactivity Disorder
Source: PLoS One. 2012 Jun 14;7(6):e39012. doi: 10.1371/journal.pone.0039012 (PMC3375242; doi:10.1371/journal.pone.0039012)
Supplement: Table S1 — Data before the calculation of lateralization (mean ± standard error). (DOC) [file pone.0039012.s002.doc]

**Supplementary table S1 – Data before the calculation of lateralization (mean ± standard error)**

|  | **ADHD** |  | **control children** |  |
| --- | --- | --- | --- | --- |
|  | C3 | C4 | C3 | C4 |
| **iMP (without MPH)** | 2.7±0.6µV | 2.1±0.5µV | 2.7±0.5µV | 4.0±0.4µV |
| **iMP (with MPH)** | 2.0±0.7µV | 2.0±0.6µV |  |  |
| **mPINV (without MPH)** | -2.6±0.4µV | -1.1±0.3µV | -1.8±0.3µV | -0.5±0.3µV |
| **mPINV (with MPH)** | -2.0±0.4µV | -1.1±0.4µV |  |  |
